# Assessment of the HBV vaccine response in a group of HIV-infected children in Morocco

**DOI:** 10.1186/s12889-017-4776-8

**Published:** 2017-09-29

**Authors:** Houda Haban, Soumia Benchekroun, Mina Sadeq, Abdelaziz Benjouad, Said Amzazi, Hicham Oumzil, Elmir Elharti

**Affiliations:** 1 National Reference Laboratory for HIV, Department of Virology, National Institute of Hygiene, Rabat, Morocco; 2 Immunology-Biochemistry Laboratory, Faculty of Sciences, University Mohammed Vth, Rabat, Morocco; 3 Pediatric Infectious Disease Clinic, Ibn Sina University Hospital, Rabat, Morocco; 4 Environmental Epidemiology Unit, National Institute of Hygiene, Rabat, Morocco; 5 International University of Rabat, Salé, Morocco

**Keywords:** HBV vaccine, HIV-infected children, Seroprotection, Morocco

## Abstract

**Background:**

Since its development in the early 1980s, Hepatitis B virus (HBV) vaccine has been proven to be highly protective. However, its immunogenicity may be ineffective among HIV-infected children. In Morocco, HBV vaccine was introduced in 1999, and since then all infants, including vertically HIV-infected infants, have been following the vaccination schedule, implemented by the Moroccan ministry of health. An assessment of the immunization of these children is important to optimize efforts aimed at tackling Hepatitis B coinfection, within the country.

**Methods:**

Forty-nine HIV-infected children (HIV group) and 112 HIV uninfected children (control group) were enrolled in this study. Samples were tested by Elisa (Monolisa Anti-HBs, Biorad) to quantify the anti-HBs antibodies. The % of lymphocyte subsets i.e. CD4+ T cells, CD8+ T cells, B cells, and NK, was determined by flow cytometry, using CellQuest Pro software (Becton-Dickinson), and for HIV group, HIV viral load was measured by real time PCR assay (Abbott). All variables were statistically compared in the two groups.

**Results:**

The median age was 51 ± 35 months for the HIV group and 50 ± 36 months (*p* > 0.05) for the control group. Female represented 63% and 41% (*p* = 0.01), among the HIV group and the control group, respectively. Among HIV-infected children, 71.4% (35/49) were under HAART therapy at the enrollment in the study. Seroprotection titer i.e. anti-HBs ≥10mUI/ml among control group was 76% (85/112), and only 29% (14/49) among the perinatally HIV-infected children (*p* < 0.0001). Lower % of CD4 + T cells was observed in HIV-infected children with a poor anti-HBs response.

**Conclusion:**

In this studied group, we have shown that despite the vaccination of HIV-children with HBV vaccine, 71% did not show any seroprotective response. These findings support the need for monitoring HBV vaccine response among HIV-infected children in Morocco, in order to revaccinate non-immunized children.

## Background

Hepatitis B still represents a global health challenge, with roughly 240 million persons are infected, and 650,000 patients die with chronic hepatitis B infection, every year [1]. Fortunately, a highly protective vaccine against HBV has been available, since early 1980s [2, 3]. Consequently, vaccination strategy against hepatitis B epidemic have become the most cost-effective public health measure implemented so far, to fight mortality and morbidity linked to this epidemic [4–6]. However, some persons may be prone to a weak response to this vaccine and therefore remain at risk of contracting HBV. For instance, in the setting of HIV infection, the vaccination does not lead necessary to immunization, since HIV-infected persons may not develop the seroprotective titer of Hepatitis B surface antibodies (anti-HBs), which is estimated at ≥10 mIU/ml [7]. Nevertheless, it was reported that the institution of HAART treatment might improve the anti-HBs response. In this context, HBV vaccine seroconversion assessment during HIV infection is important to evaluate whether these children are seroprotected or need extra doses to augment the probability of their immunization [8, 9].

Morocco is considered as an intermediate country of endemicity of hepatitis B, since the prevalence of hepatitis B infection was estimated at 1.8% across the country, in 2011 [10]. However, the prevalence varied widely in different groups. For example, in blood donors, it was around 1% in 2010, whereas in other groups like barbers, it was much higher with a rate of 28% [11, 12]. To curb the epidemic, the Moroccan ministry of health introduced the HBV vaccine in 1999. Since then, the vaccine has been provided by the national program of immunization to all infants, including those infected with the HIV virus. The national vaccine schedule encompasses three doses given at 2, 3 and 4 months after birth. In 2015, another dose at birth was added.

The objectives of this study were to compare the vaccine response among perinatally HIV-infected children and HIV non-infected children, and to determine the prevalence of protective vaccine titer for both groups.

## Methods

Children visiting Ibn Sina Children’s University Hospital in Rabat, between January 13, 2011 and June 16, 2013 were the target population. A total of 53 children already known to have HIV, and 118 HIV-free children visited the hospital during the study period, for respiratory diseases (control group). Control group children were tested negative for HIV, by a rapid test (HIV Determine, Alere). A total of 10 children were excluded as there was not enough specimen for laboratory tests. Children from both groups have to be aged between 10 months to 10 years old, and should have completed the national HBV vaccine schedule. This could be known from the individual vaccination record book. Having primary immunodeficiency diseases or hepatitis B was an exclusion criterion of children, for both groups. Parents or guardians of children participating in this study signed a written informed consent prior to any enrollment. This study received the ethical approval from the Ethic Committee of Biomedical Research, Medical School and Pharmacy, University Mohammed V^th^, Rabat, Morocco.

### Laboratory tests

Serological tests: All samples were tested by ELISA assay to quantitate the titer of anti-HBs (Monolisa Anti-HBs Plus, Biorad, France). To roll out hepatitis B infection, all negative samples for anti-HBs antibodies are further analyzed for Hepatitis B surface antigen (HBsAg) by ELISA testing (Monolisa AgHBs Ultra, Biorad, France) and for Hepatitis B core antibodies (anti-HBc) (Monolisa Anti-HBc Plus, Biorad, France).

Flow Cytometry: The percent of lymphocyte subsets i.e. T cells, CD4+ T Cells, CD8+ T cells, CD19+ cells (B cells) and CD16+/CD56+ cells (NK cells), was determined by performing immunostaining and analysis on flow cytometry, using CellQuest Pro (Becton-Dickinson, USA).

Automated Real time PCR: HIV viral load was measured by using the Abbott Real Time HIV viral load Assay (Abbott, USA). RNA quantitation was expressed in log10.

Regarding lymphocytes subset measurement, samples were tested within 4 h after blood collection, however for HBV testing and HIV viral load assay, samples were frozen at −20 °C and tested in batches.

All laboratory analyses were conducted at the National Reference Laboratory for HIV, National Institute of Hygiene, in Rabat.

### Statistical analysis

Data were entered into an Excel sheet and descriptive analysis was first performed to provide arithmetic mean, standard deviation, and geometric mean for different set of data. A 2 by 2 chi-square test was also performed to compare data between the HIV-infected group and the control group. A spread of data on anti-HBs response (log10 scale) across both groups was given. We also compared medians of variables, including T cells, CD8+ T cells, CD4+ T cells (scores), NK cells and B cells, for a protective response against HBV vaccine for both groups, using Mann–Whitney U test: for a lower side (respectively an upper side) test the alternative hypothesis is that values related to HIV-infected group tend to be smaller (respectively larger) than those related to the control group. Data was considered significative at *p* < 0.05. All statistical analyses were done using the StatsDirect statistical software version 3.0124 (StatsDirect, Cheshire, UK).

## Result

A total of 49 HIV-infected children and 112 controls participated in this study. Table 1 shows clinical data related to these two groups. Mean age was 51 ± 35 and 50 ± 36 months for the HIV group and the control group, respectively. Observations in the two groups were similarly distributed (median difference = 0 and 95% confidence interval for difference between medians = [−12 and 6]. Female represented 41% and 63% in the control group and the HIV group, respectively: the proportion difference = 0.22 and the two-sided *p*-value = 0.01. Among HIV-infected children, HIV viral load was 2.4 ± 2. log. Furthermore, out of 49 HIV-infected children, 35 (71.4%) were under HAART therapy, whereas 14 patients were not. In this study, only B cells and NK cells in the HIV-infected group tended to be less than those in the control group (*p* < 0.0001). Regarding CD4+ cells, the HIV-infected group did not tend to yield different CD4+ T cells scores to the control group (*p* = 0.703).

**Table 1:** Clinical data of studied children

	Control group m ± SD	HIV-infected group m ± SD	Median difference	95% CI for medians difference
% T cells	64.1 ± 9.8	76.7 ± 7.3	12	[9, 15]^b^
% CD8+ T cells	25.4 ± 7.7	44.3 ± 12.0	19	[15, 22]^b^
% CD4+ T cells (scores)	34.9 ± 6.9	25.6 ± 9.2	0.10^a^	[−0.26, 0.43]^b^
% B cells	20.7 ± 8.9	14.8 ± 7.2	−6	[−9, −4]^a^
% NK cells	09.0 ± 5.6	5.76 ± 3.3	- 3	[−4, −1]^a^

^a^the research hypothesis is supported *p* < 0.0001

^b^the research hypothesis is not supported

**Fig. 1:**
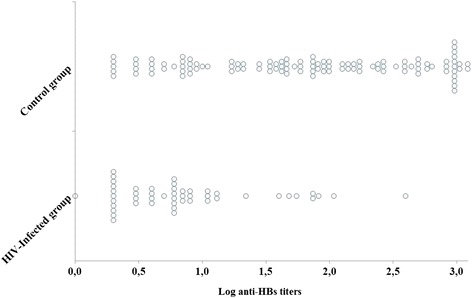
HBV vaccine response in the HIV-infected group and the control group

Vaccination response is presented in Table 2. The geometric mean titer of anti-HBs among the control group was 609.5, whereas it was 142.5 among HIV-infected children. During this study, 76% (85/112) children from the control group had anti-HBs ≥10 mUI/ml, but only 29% (14/49) among the HIV group developed a similar level of antibody response (*p* < 0.0001). Figure 1 provides a pictorial representation of the spread of observations related to anti-HBs antibody across groups. A concentration of data at particular value is represented by a broad band. Regarding the control group, 42% (47/112) developed a protective antibody anti-HBs level higher than 100 mIU/ml, whereas only 4.1% (2/49) in the HIV group reached such level of antibody anti-HBs (data not shown). All children with negative anti-HBs result were negative for HBsAg and anti-HBc as well.

**Table 2:** HBV vaccine response among studied children

	Control group	HIV-infected group	*p*-Value
Children with protective anti-HBs response	85 (76%)	14 (29%)	<0.0001
Children without protective anti-HBs response	27 (24%)	35 (71%)	<0.0001
Geometric mean level	609.46	142.53	–

When comparing patients that developed protective antibody titer greater than 10 mUI/ml, and children with antibody anti-HBs level lower than 10 mUI/ml (Table 3), in the control group; suboptimal HBV vaccine response was associated with a relatively lower percent of B cells (*p* = 0.029). Nevertheless, in the HIV-infected group, the percent of CD4+ T cells is lower in children with inadequate anti-HBs antibody response (*p* = 0.008). Moreover, 85.7% of seroprotected patients were under HAART treatment, while 65.7% were not, but the difference was not statistically significant (*p* = 0.185). Finally, HIV viral load was 2.4 ± 2.3 log and 2.4 ± 2.1 log, for children with optimal vaccine response and children with inadequate vaccine response, respectively (*p* = 0.5).

**Table 3:** Data by HBV vaccine response

	Control group			HIV-infected group		
	Anti-HBs < 10	Anti-HBs ≥10	*p*-Value	Anti-HBs < 10	Anti-HBs ≥10	*p*-Value
	(*n* = 27)	(*n* = 85)		(*n* = 35)	(*n* = 14)	
	m ± SD	m ± SD		m ± SD	m ± SD	m ± SD
Age (months)	64 ± 38	45 ± 35	0.991	59 ± 36	32 ± 26	0.998
Female	56%	37%	0.080	69%	50%	0.223
%T cells	67.5 ± 11.5	63.1 ± 9.0	0.986	75.7 ± 7.8	75.6 ± 5.9	0.652
%CD4+ T cells	34.1 ± 6.7	35.1 ± 7.0	0.741	23.6 ± 9.0	30.4 ± 8.4	0.008
%CD8+ T cells	27.4 ± 7.5	24.8 ± 7.6	0.963	46.0 ± 12.6	40.1 ± 9.2	0.928
%B cells	17.9 ± 9.5	21.6 ± 8.5	0.029	14.0 ± 7.0	16.7 ± 7.3	0.056
%NK cells	8.9 ± 5.0	09.1 ± 5.8	0.593	06.3 ± 3.1	04.3 ± 3.5	0.983
HAART	NA	NA	NA	65.71%	85.71%	0.185
HIV Viral load (log)	NA	NA	NA	2.4 ± 2.3	2.4 ± 2.1	0.5

*m* arithmetic mean, *SD* standard deviation, *NA* not applicable

## Discussion

This is the first study that assesses the HBV vaccine response in a group of children with vertically acquired HIV infection, in Morocco. The prevalence of protective HBV vaccine response among the control group is 76%, whereas it does not exceed 29% among HIV-infected children (*p* < 0.0001). This result is in accordance with previous studies that have reported similar low vaccine responsiveness, in children living with HIV/AIDS [13, 14]. In addition, our findings show that HIV-infected children with a poor HBV vaccine response had a lower percent of CD4+ T cells, when compared to children with a higher protective level of anti-HBs antibodies. This result is consistent with other studies [15, 16]. In fact, during HIV infection, viral replication damages the immune system to the extent that the response to HBV vaccine becomes suboptimal or inexistent, and children may remain at risk of contracting HBV infection [17, 18]. Luckily, with the advent of HAART therapy, viral multiplication can be suppressed and the immune system is therefore restored. Consequently, the HBV vaccine response can be improved for these children [19, 20].

The poor responsiveness to HBV vaccine among the HIV children, observed in this study may also be explained by a faster decline of vaccine titer after the initial response [21].

In this study, even though the percent of patients under HAART with suboptimal anti-HBs response is lower compared to children with a protective level, the difference is not statistically significant. Furthermore, HIV viral load was not different for children with a protective vaccine response and those with impaired response. These results may be explained by the small size of the sample. In addition, some patients may experience a limited immune restoration and then develop a weak vaccine response, despite the HAART treatment [22, 23].

We report in this study that the HBV vaccine response is limited among the HIV-infected children. Additionally, vaccine antibody titer higher than 100 mIU/ml was observed in 42% in the control group, whereas only 4% of HIV-infected children showed a similar anti-HBs titer. Although the level of anti-HBs antibodies considered to confer protection is estimated at ≥10 mIU/ml, a higher titer is pivotal for a sustainable protective response, since lower antibody levels tend to wane rapidly, over time [24].

There are several study limitations to be considered when evaluating our findings. First, the small, non-probability sample of convenience for the HIV infected children, as this number cannot reflect the reality of this population, in Morocco. Second, the duration of HAART therapy for HIV-infected children at the enrollment in the study, is missing. Third, children with seroprotective anti-HBs titer, are not tested for HBc antibodies and therefore the number of children with natural immunization was unknown. Fourth, at the time of vaccination CD4+ T cells count as well as suppressed viral load are not known. Fifth, the time of HAART initiation in respect to the vaccination is missing. Finally, there are more females among the HIV group.

Despite these limitations, the findings represent a key baseline information about HBV vaccination response in Morocco, for the national program of immunization. In addition, the results suggest that HIV-infected children may not be immunized against hepatitis B, despite the vaccination. Therefore, the assessment of HBV vaccine response, after vaccine completion, should be recommended. This will help identify children that require an extra cycle of vaccination. These measures may limit the risk of the coinfection with HBV infection, as recommended by international guidelines, which advocate clearly that HIV-infected children with a low or no seroconversion should benefit from another cycle of vaccination, in order to increase the odds of getting an adequate protection against HBV [25–27].

## Conclusion

In this study, we have shown that most of vertically HIV-infected children studied develop a suboptimal response to HBV vaccine. Therefore, post-vaccination monitoring of the anti-HBs seroconversion is fundamental, for HIV-infected children. This will help optimize vaccination against hepatitis B, for these patients, across the country.

## Abbreviations

Anti-HBs: Hepatitis B surface antibodies
HAART: Highly Active Antiretroviral Therapy
HBV: Hepatitis B virus
